# Quality control protocol of the National Mental Health Survey-2 of India

**DOI:** 10.1371/journal.pone.0355561

**Published:** 2026-08-20

**Authors:** Girish N. Rao, Vrunda A. Patel, NMHS-2 Central Monitoring Team, Central Advisory Group, Pratima Murthy, Vivek Benegal, T. S. Jaisoorya

**Affiliations:** 1 Department of Epidemiology, National Institute of Mental Health and Neurosciences (NIMHANS), Bengaluru, Karnataka, India; 2 Department of Psychiatry, National Institute of Mental Health and Neurosciences (NIMHANS), Bengaluru, Karnataka, India; 3 National Institute of Mental Health and Neurosciences (NIMHANS), Bengaluru, Karnataka, India; PLOS: Public Library of Science, UNITED KINGDOM OF GREAT BRITAIN AND NORTHERN IRELAND

## Abstract

The National Mental Health Survey of India – 2 (NMHS-2), currently underway (2024–26), is one of the largest psychiatric epidemiological studies globally, interviewing more than 250,000 individuals across all 36 states and union territories. Given India’s scale and diversity, a rigorous quality control (QC) protocol has been developed to ensure methodological fidelity, data reliability, and operational consistency. Designed through iterative consultations with national and international experts, and approved by the National Technical Advisory Group, the protocol leverages lessons from NMHS-1 and integrates global best practices to meet India’s unique challenges. This protocol aims to describe the QC framework of NMHS-2, highlighting its design, core components, and implementation strategies. Key components include a multi-tiered administrative structure, systematic instrument selection, its validation and translation; structured training programs for field data collectors; and pre-survey pilot testing of the sampling design. The critical Core QC strategy involves a custom-made digital data collection platform with built-in logic checks, real-time dashboards, multi-source data verification, and re-interviews, apart from standardized data cleaning protocols. Comprehensive documentation and regular review meetings at both state and central levels further strengthen accountability and transparency. By integrating international standards with locally tailored approaches, NMHS-2 establishes a replicable model for quality assurance in large-scale mental health surveys. This paper addresses a critical gap in the literature on structured QC protocols for psychiatric epidemiological studies.

## Introduction

The National Mental Health Survey of India – 2 (NMHS-2), funded by the Ministry of Health and Family Welfare, Government of India, is currently underway (2024−26) and stands as one of the largest global epidemiological surveys of mental morbidity. Once completed, the survey will have assessed an estimated >250,000 individuals across all 36 Indian states (n = 28) and union territories (n = 8). The survey employs a rigorous methodology, refined through multiple layers of scientific iterations before its acceptance by the National Technical Advisory Group (NTAG) (under publication). Even in rigorously designed, methodologically sound studies, well-structured quality control (QC) mechanisms are critical to ensure that scientific integrity is not compromised due to procedural deviations, interview-related variability, or inconsistencies in field implementation [1].

Mental health surveys face more significant challenges, and QC measures are exceptionally critical. Unlike many physical conditions, there are no objective biomarkers for mental health; mental health assessments depend on self-reports, which can be variably interpreted by both the respondent and assessors. Hence, quality control remains a critical aspect. However, Quality control protocols are underreported and are often poorly documented components of large-scale mental health surveys. The World Mental Health (WMH) Survey Initiative played a leading role in standardizing psychiatric epidemiology across a range of countries and diverse contexts [2,3]. The relatively few publications from the WMH initiative have focused explicitly on the design and implementation of QC frameworks, despite their critical importance in large-scale field surveys. Recent reports from the Saudi and Qatar National Mental Health Surveys are exceptions [4,5].

The QC protocol for NMHS-2 is meticulously predefined, considering the complexities of conducting a mental health survey across India’s diverse geographic, linguistic, and cultural landscapes. This paper details the QC protocol in NMHS-2 and outlines its core components. It presents the multi-tier governance framework, detailed processes for instrument selection, validation, and translation, the digital systems used for data collection and monitoring fieldwork, the procedures for training and supervision, and data management and cleaning. Documenting these processes is crucial for transparency for the NMHS-2. It also addresses a significant gap in the literature by offering a practical, adaptable model for quality assurance in mental health surveys—especially in resource-constrained settings.

## Methodology

### Unique challenges for NMHS-2 from a quality control perspective

A core group comprising senior Indian psychiatrists and epidemiologists, all with prior involvement in large mental health surveys, including the National Mental Health Survey-1 (NMHS-1) of India, was constituted to identify potential challenges in NMHS-2 from a quality-control perspective. This team identified a multitude of QC challenges through a meticulous review of experience with NMHS-1, other major surveys, and published QC protocols.

The 6 anticipated challenges broadly identified are briefly described below-

**Administrative framework**: Develop a multi-tiered administrative framework to effectively coordinate, ensure accountability, and maintain consistency across various survey sites of the NMHS-2.

**Instruments for assessment**: Selecting appropriate survey instruments for assessment and ensuring their cultural adaptation and local validation before administration.

**Training**: Develop and implement a standardized, uniform training program for field data collectors (FDCs). This training, which bridges skills gaps among the FDCs’ educational backgrounds and field experience, also addresses the limited availability of qualified trainers in select states. It needs to be inherently adaptable to the demands of administering surveys in 20 different languages across a variety of settings.

**Pilot testing**: Mandated across all sites to ensure that the survey methodology is uniformly implemented while aligned with the specific state’s needs. This process will overcome logistical challenges and factor variations in mental health literacy and address community-level stigma.

**Field-level monitoring**: Operationalizing effective field-level monitoring ensures data integrity, protocol adherence, and timely troubleshooting of field-level challenges.

**Data collection platform and data management**: Developing an online platform to empower data collection, especially supporting the use of multiple languages, and enabling offline use. Data management needs to ensure structured data workflows, including near real-time digital data entry, and support rigorous cleaning protocols Table 1.

**Table 1:** Anticipated QC challenges and corresponding strategies.

Anticipated QC Challenge	Strategy Adopted
Preparatory stage	
Coordinating a large, multi-state survey and ensuring consistency, accountability, and oversight across 36 states and UTs	Establish a multi-tiered administrative framework with central, local, and advisory levels for implementation, supervision, and support
Selecting appropriate instruments suitable for lay interviewers in diverse settings	Use structured, ICD-11 compatible tools that are culturally adapted and locally validated before use. Follow WHO-aligned translation–back-translation with expert and state-level review.
Bridging variability in the educational background, skills, and language in FDCs across diverse regions	Training through a cascading model. Implement a standardized 6-week training program with detailed centralized SOPs, supervised practice, and an end-of-training evaluation for FDCs; In-house training by the central team for states with human resource constraints. Moodle-based learning support.
Handling constraints in digital connectivity, infrastructure, reduce interviewer related errors, and maintaining data quality across regions	Use a tablet application that can work offline on a custom-built multilingual digital platform, with built-in logic checks, geo-tagged entries, syncing to a central server, and clear instructions on data handling.
Data collection stage	
Ensuring feasibility and operational readiness in states with very different resources	Pre-survey pilot testing in every state for a minimum of one week to refine/check logistics, interview skills, and field procedures
Maintaining protocol adherence, methodological fidelity, and data integrity during fieldwork	Use structured field monitoring steps, including daily logs, cluster summaries, and supervisory review at state and central levels
Preventing and detecting interviewer-related variability or data falsification	Monitor digital paradata (e.g., interview duration, GPS codes, detection rates), conduct re-interviews and verification calls
Ensuring transparent documentation	Ensuring fidelity to pre-defined SOPs, monitoring records, and centralized documentation for all steps of the survey for all states
Post-data collection stage	
Managing and cleaning a large, complex multi-state dataset consistently	Apply a scripted, version-controlled data management and cleaning protocol with predefined checks and documentation

This table summarizes anticipated quality control challenges and the strategies implemented to address them across different phases of the survey.

### Quality control protocol of the NMHS-2

The central coordination team at the (Institution name withheld for blinding) initially developed the QC protocols for NMHS-2 to address the anticipated challenges detailed above. The initial draft protocol was meticulously reviewed and modified by a consensus of senior faculty and state collaborators from all States and Union Territories to ensure its nationwide applicability. The National Technical Advisory Group of NMHS-2 examined this draft and provided suggestions that were incorporated.

**Fig 1:**
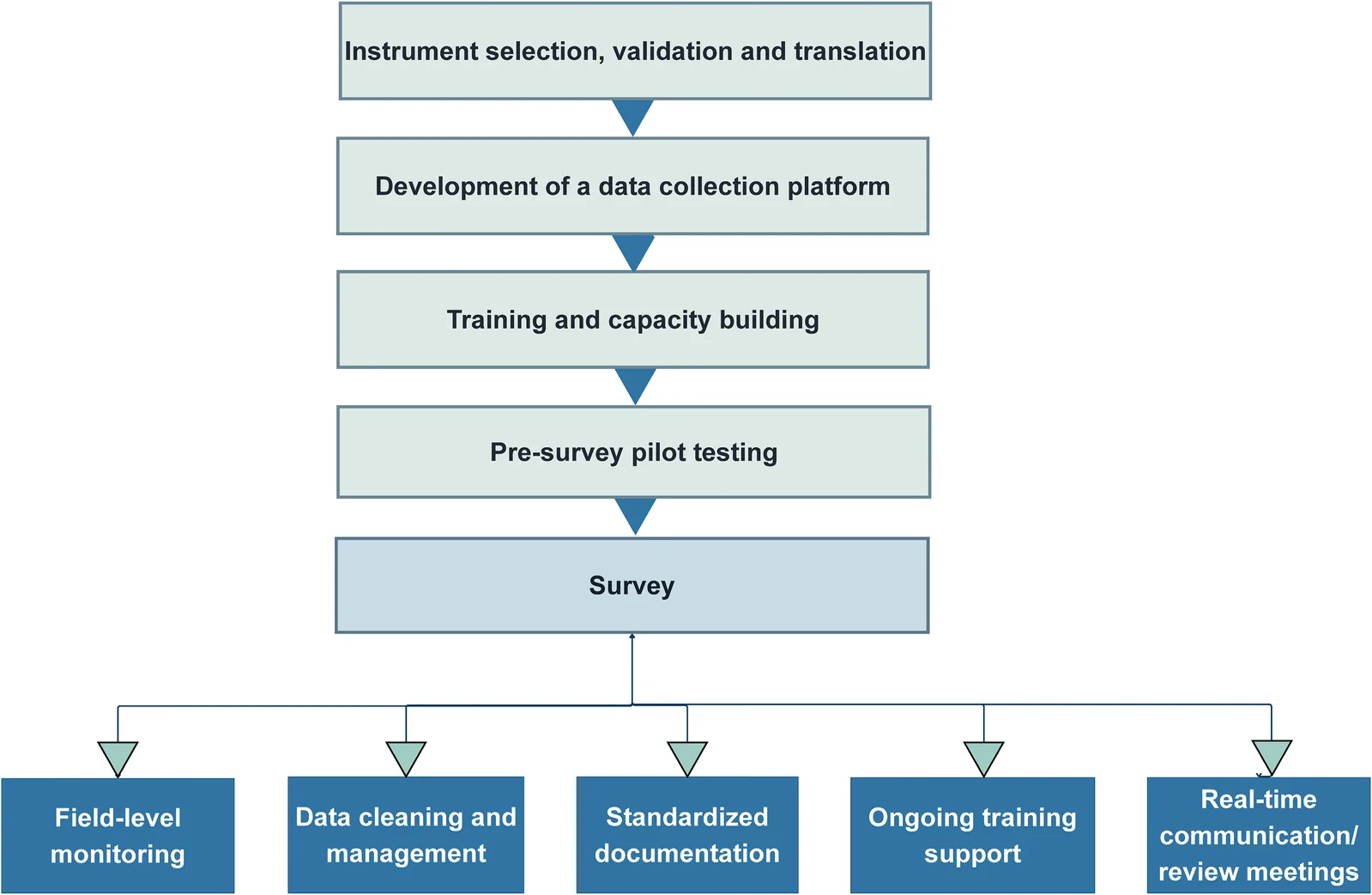
Comprehensive quality control protocol of NMHS-2. The figure presents the overall quality control protocol of the NMHS-2, illustrating the key domains and the interlinkages between these components across different phases of the survey.

**Fig 2:**
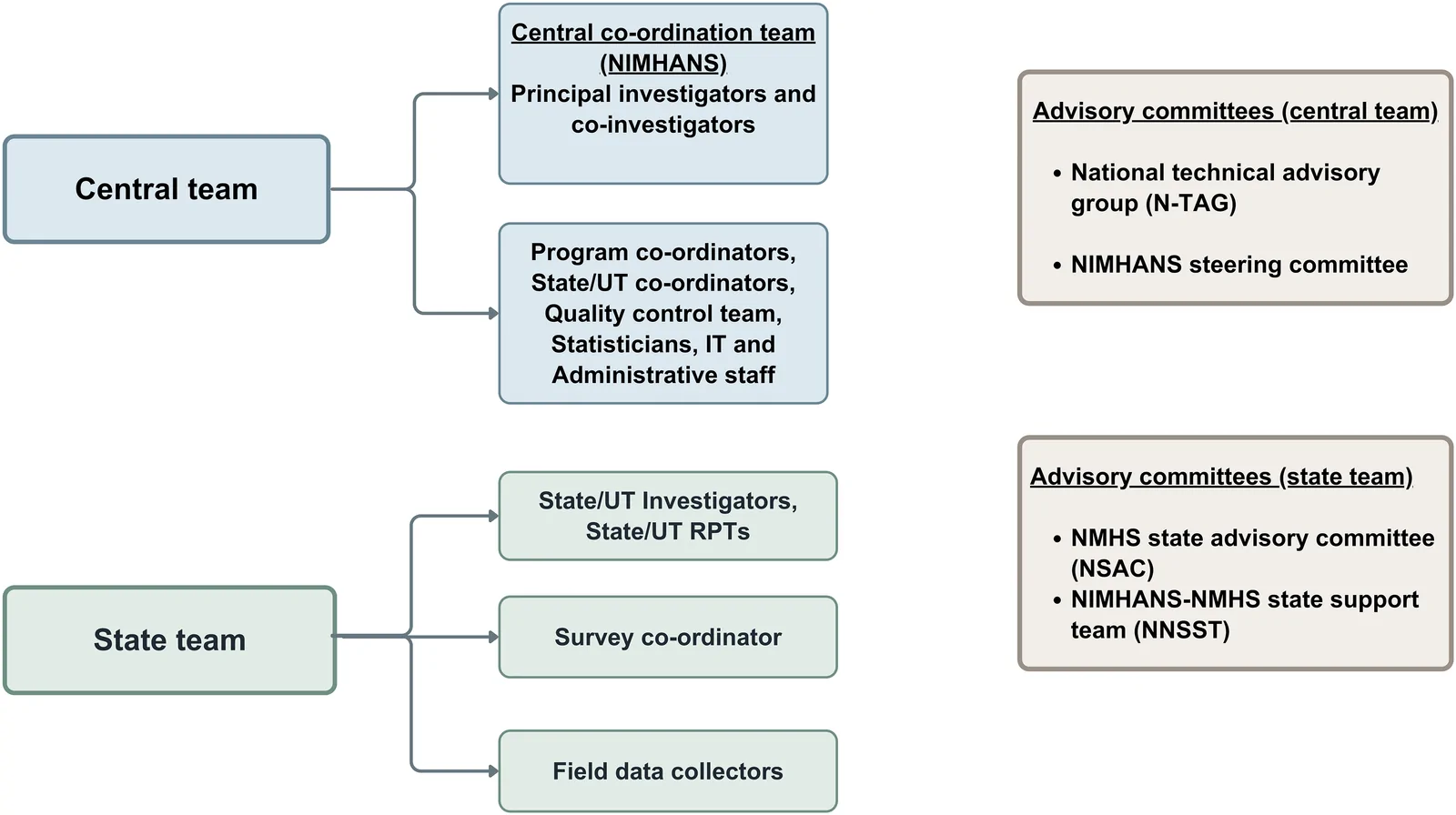
Operational Structure of NMHS-2. The figure depicts the multi-tiered administrative and supervisory structure of NMHS-2.

**Fig 3:**
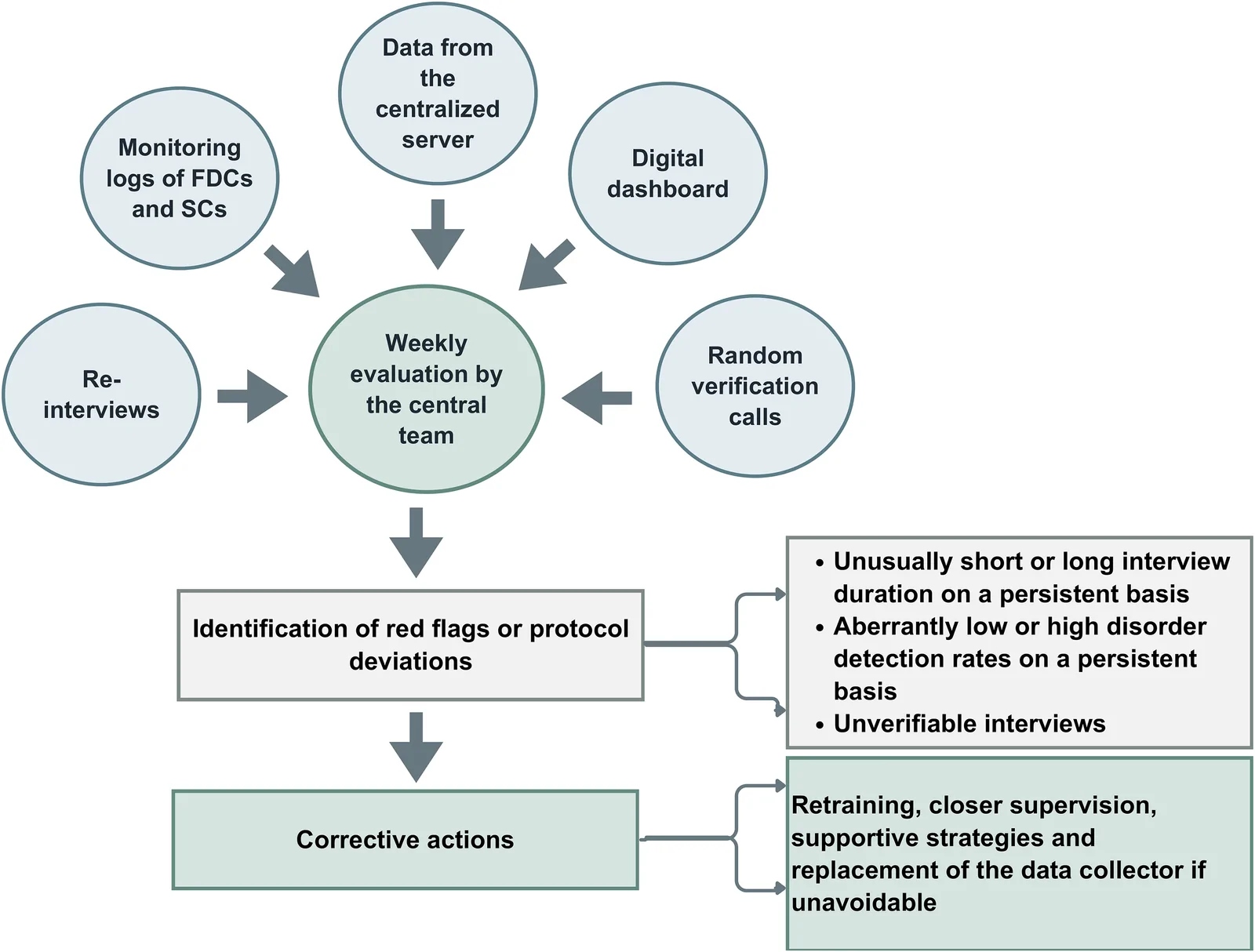
Field-Level Monitoring. The figure outlines the workflow for field-level monitoring and QC processes during the survey. FDC- Field data collector, SC-Survey co-ordinator.

The core components of the QC protocol of the NMHS-2 are detailed below Figs 1–3.

**1. Multi-tiered administrative framework:**

A multi-tiered supervisory framework is essential for quality control, given the need to standardize assessments of a large number of individuals representing diverse geographic, linguistic, cultural, and demographic backgrounds over an extended survey period. The NMHS-2 administrative framework comprises field-level personnel, survey coordinators, state study teams, central coordinating units, and advisory bodies, each with defined responsibilities. Field data collectors are responsible for standardized data collection and documentation, while survey coordinators (SC), in addition to undertaking data collection, supervise field activities and facilitate communication and coordination between the field team and the state and central coordinating units. State study teams support recruitment of field teams, training, monitoring field performance, and addressing implementation challenges. The Central team provides technical guidance, reviews quality indicators, and monitors survey progress across sites, while advisory bodies periodically review survey processes and provide expert recommendations and strategic guidance. Together, this hierarchical framework supports quality control through clearly defined channels for supervision, reporting, escalation, and resolution of operational issues identified during survey implementation. Such mechanisms are particularly important in large-scale, multi-site surveys to ensure timely corrective action and maintain procedural standardization across sites. This strategic approach has also been previously employed in other large-scale mental health surveys, including the NMHS-1 [6]. (Detailed descriptions of the composition and overall roles of these bodies are provided in the NMHS-2 Methodological Framework paper.)

**2. Instrument selection, validation, and translation:**

The NMHS-2 has two key objectives: the first is to estimate the prevalence of mental health morbidity, and the second is to assess the disability due to mental morbidity. Since the survey was to be administered by trained lay interviewers, additional considerations in instrument selection included ensuring that the tools were short, easy to use, and structured with a fixed script to allow direct recording of the respondent’s answers without the need for the interviewer’s interpretation. To ensure the collected data were valid and reliable, the chosen instruments were culturally adapted and examined for their psychometric properties within our population.

The Flexible Interview for ICD-11 (FLII-11), a WHO structured diagnostic interview, was selected as the primary diagnostic instrument for NMHS-2 due to its compatibility with ICD-11 criteria and suitability for use by trained lay interviewers [7]. As the FLII-11 was new, prior to the survey onset, the instrument underwent cultural adaptation. The culturally adapted FLII-11 was then clinically validated, with results showing strong psychometric properties (κ = 0.81, sensitivity = 83.3%, and specificity = 78.8%) [8]. An instrument to assess disability has also been developed in the NMHS-2 (NIMHANS-NMHS2-DisabilityScale), which showed a strong correlation with the gold standard – the 12-item WHO disability assessment schedule 2.0 (rho = 0.93) [9].

Other supplementary instruments that have been included have been developed or adapted to address the population-specific needs of Indians. These include tools for screening neurocognitive disorders (Screening tool for ICD-11 Dementia in epidemiological studies [SIDES]) and capturing parental concerns in children (Checklist of Parental Concerns). (manuscript submitted for publication elsewhere) Instruments measuring caregiver and family burden and stigma at both individual and community levels were adapted from existing validated scales, while modules on healthcare utilization and barriers to accessing services were revised from NMHS-1. These tools underwent validation in clinical and control samples, demonstrating acceptable reliability and diagnostic performance. (Detailed descriptions of all the instruments used in the survey are provided in the NMHS-2 Methodological Framework paper.)

All instruments were translated into 20 Indian languages following the WHO translation protocol [10]. Initial drafts prepared by a professional agency were reviewed by bilingual mental health professionals during centralized workshops at (Institution name withheld for blinding). State teams subsequently assessed the translations for cultural and linguistic appropriateness, and an independent agency conducted back-translations. Final reconciliations ensured semantic fidelity and contextual relevance across diverse linguistic regions.

**3. Training and capacity building:**

Given the reliance on trained lay interviewers for data collection, standardized and uniform training is considered a critical component of the NMHS-2 quality control framework. The objectives of training are to ensure uniform understanding of survey procedures, minimize interviewer-related variability, and promote consistent administration of study instruments across all participating States and Union Territories.

**3.1 Training of trainers workshops:** Training for Principal Investigators and Co-Investigators from all states and UTs was conducted over two days in Training-of-Trainers workshops by the central team at (Institution name withheld for blinding). These sessions covered survey methodology, instrument use, field protocols, budgetary aspects, and quality control procedures. This was to facilitate structured knowledge transfer through a cascading model and ensure standardization and fidelity of training across sites.
**3.2 Resource persons for training (RPT):** Additionally, online sessions are being held for state-level RPTs as required to strengthen each state’s capacity to supervise, train, and mentor FDCs throughout the survey to maintain uniform interpretation and delivery of training procedures across sites.
**3.3 Structured training for field data collectors:** To ensure uniformity across diverse regions, the central team developed a detailed SOP to guide the six-week training program for FDCs, which is conducted locally by state teams in the respective regional languages. State teams recruit FDCs who meet specific criteria: a basic knowledge of mental health, reflected by an academic background in psychology, social work, public health, or nursing; fluency in the local language; and, preferably, prior experience in mental health settings. Training includes lectures, video demonstrations, role plays, and supervised field practice in both clinical and community settings. FDCs are formally evaluated and certified prior to deployment. A faculty from (Institution name withheld for blinding) Behavioral Science Division visits each state at the end of training to assess field readiness and review training quality. A centralized Moodle platform, managed by the central team, hosts all survey materials, including instructional videos and tools to support continuous asynchronous learning and ensure consistent training delivery across the country.
**3.4 Intensive centralized training:** A few states with limited training capacity due to human resource constraints require extra support for training FDCs. To address this, a two-week intensive in-house training program is being conducted at (Institution name withheld for blinding), focusing on conceptual and theoretical aspects of the survey. The FDCs then undergo supervised observation and hands-on practice in their respective states. This training is also beneficial for states that need to recruit and train new FDCs to replace those who resign mid-way during the survey.

**4. Pre-survey pilot testing:**

Upon completion of training, each state conducts the pilot survey for at least one week, or until field readiness is achieved. Field readiness is determined based on predefined criteria, including correct implementation of the sampling protocol, satisfactory interview techniques and stable interview durations, absence of major protocol deviations identified by state investigators, and acceptable performance indicators monitored by the central team. Pilot testing also facilitates identification of state-specific issues related to language, terrain, connectivity, logistics, and community engagement, allowing remedial actions to be undertaken before full-scale data collection. Insights from the pilot also help resolve logistical and training-related challenges, enhancing consistency and preparedness across sites. The pilot is undertaken in an area not selected for the main survey and includes both urban and rural locales. Only teams that demonstrated satisfactory field readiness during pilot testing were permitted to proceed to the main survey.

**5. Data collection platform: design and quality features:**

The NMHS-2 digital data collection platform is designed to support multiple quality assurance functions during survey implementation [11]. The software supports multi-language input across 20 Indian languages and offline functionality to ensure uninterrupted data entry in poor/ low-connectivity areas [12]. Built-in logic checks, including skip patterns and response validations, reduce data entry errors in real time. Role-based access controls were implemented to ensure data security and restrict modifications to authorized personnel. GPS-enabled geotagging supported verification of field locations and adherence to sampling procedures. The platform also enabled centralized monitoring through real-time dashboards that tracked survey progress and key operational indicators across participating States and Union Territories. The application was rigorously tested using clinical vignettes prior to nationwide deployment to ensure consistency with quality control protocols.

**6. Field-level monitoring:**
  **6.1 Digital dashboard:** A centralized digital dashboard has been created for real-time monitoring of key metrics—such as completed interviews, cluster status, and gender-wise distribution—across all states and union territories, supporting timely oversight by the central team [13].
  **6.2 Field data monitoring:** Effective field-level monitoring is a cornerstone of quality assurance in large-scale epidemiological surveys [14] In NMHS-2, this is operationalized through a structured system that ensures data integrity, protocol adherence, and timely identification of field-level challenges.

The protocol followed for field-level monitoring is to ensure triangulation of data from multiple sources by the central team, and involves the following data sources:

Primary data source: FDCs sync collected data to a secure central server daily. In areas with limited internet connectivity, data syncing is deferred until access becomes available. This data, synced by the FDC to the central server at (Institution name withheld for blinding), forms the primary data source.

Secondary data sources: Daily logs of FDCs: FDCs to maintain daily logs documenting household visits, completed interviews, refusals, and revisits.

Summary logs of FDCs**:** The Survey Coordinator is required to consolidate the daily logs of all FDCs into daily and weekly summaries.

Cluster summary form: Upon completing each sampled cluster, the survey coordinator also completes a Cluster Summary Form detailing the number of households approached, interviews conducted, individual and household refusals, and field observations.

The key strategy for field-level monitoring is to examine patterns of deviation of data information from the primary source, which is the data uploaded to the server, and the information received from the secondary sources of information of the FDCs and the survey coordinator.

**6.3 Digital performance monitoring:** Continuous monitoring of field data collectors’ performance is known to reduce measurement error and interviewer-related variability [15,16]. FDCs are monitored against a set of predefined performance indicators. Potential concerns or “red flags” include unusually short interview durations (i.e., less than 20 minutes, based on pilot testing by central team Psychiatrists), aberrantly high or low disorder detection rates, and unverifiable interviews. Random telephonic callbacks by the QC team to a subset of respondents serve to verify the interviewer’s presence and assess the duration of interactions. Built-in GPS geo-tagging within the data collection tablets further ensures fidelity to sampling locations.

In practice, as the survey progressed, it became clear that connectivity-related challenges could prevent the definition of rigid action thresholds for one or more parameters. For instance, reliance on ‘interview duration’ as a standalone metric was constrained because FDCs in rural areas could not upload data in real time, or verification callbacks were often impossible in remote areas. Consequently, quality assessment relied on data triangulation. This approach proved more meaningful by cross-referencing intra-team variations (such as aberrantly high or low disorder detection rates) with data on unverifiable interviews and assessment timings. When consistent deviations are identified, remedial action should be initiated. This includes discussions with State PIs for additional monitoring, specific retraining where required, and—in very exceptional cases—termination of the field data collector.

**6.4 Sampling protocol monitoring:** Cluster maps are included to verify adherence to sampling protocols, followed by district and state summary forms upon completion of respective units. All monitoring forms are maintained both as hard copies and in Google Sheets, enabling real-time access and review by the central quality control team at (Institution name withheld for blinding). This dual system enables both immediate oversight and long-term documentation, ensuring consistent field implementation across states.
**6.5 Reinterviews:** To assess the consistency and reliability of data collection, a standardized re-interview protocol for the FLII-11 and the disability scale, which are the primary assessment instruments, is implemented across all states. Re-interviews are conducted concurrently by the expert and the FDC (joint inter-rater reliability). The expert observes the interview and doesn’t administer the instruments independently; hence, the process is not blinded. Each FDC is evaluated in a random, rotational manner, and structured, individualized feedback is provided to improve interview quality. A uniform target of 200 reinterviews per state has been adopted, based on 5% of the total sample size in the smallest participating state, to ensure feasibility across states with varying resources. These have been distributed as follows: 150 re-interviews to be conducted by Survey Coordinators, 50 by trained Resource Persons for Training—psychiatrists or community medicine faculty who have received dedicated instruction in administering the FLII-11. Re-interviews by central team psychiatrists are conducted if language compatibility permits. Findings from re-interview procedures are periodically reviewed to assess consistency of instrument administration and identify potential interviewer-related variability requiring corrective action.
**6.6 Real-time communication:** All stakeholders remain connected through dedicated WhatsApp groups, where the central team shares daily national updates on interviews completed, clusters covered, and households contacted. State Survey Coordinators also post weekly progress reports. Important announcements and survey-related updates are also communicated in these groups. Alongside email communication, this system ensures rapid information sharing, prompt resolution of field challenges, and consistent adherence to survey protocols.

**7. Refresher and remedial training:**

The central team conducts monthly online refresher training sessions of about ten hours for all FDCs. When an FDC shows poor re-interview concordance, or other QC deviations, retraining is provided in a stepwise manner—locally when possible, through onsite support from central team psychiatrists when needed, or, in selected cases, through a two-week intensive in-house program at (Institution name withheld for blinding). Additional online sessions are also provided when state-specific concerns arise.

**8. Data management and cleaning:**

Data collected through the digital platform are synchronized to a central server and undergo systematic review using predefined data-management procedures. Working datasets are cleaned using SPSS v29, following a structured data dictionary covering over 1,700 variables. The cleaning protocol includes verification of variable attributes, range and consistency checks, skip-pattern validation, duplicate record detection, and assessment of missing data. Records identified during quality review are verified against source data and resolved in consultation with field teams whenever feasible. The extent and pattern of missing or inconsistent data are periodically evaluated, and unresolved discrepancies are managed according to predefined criteria established prior to analysis.

All cleaning procedures are fully scripted, version-controlled, and documented to ensure reproducibility, transparency, auditability, and future analytical use [17]. An anonymized, write-protected Master Cleaned Dataset is finalized following multiple rounds of quality checks for analysis.

**9. Standardized documentation:**

To promote consistent implementation of survey procedures, the central team developed comprehensive standard operating procedures (SOPs) covering all major aspects of survey conduct, including field operations, re-interviews, data management, information technology systems, referral pathways for participants in distress and child sexual abuse cases, survey closure procedures, management of unreturned survey materials and devices, budget administration, and quality assurance activities. These SOPs are shared across all survey sites.

Each state maintains organized documentation, including ethical approvals, MoUs, consent forms, and monitoring logs, serving as a local record of compliance and progress, and is to be reviewed by the external monitor. In parallel, central program coordinators compile weekly monitoring reports and maintain state-specific folders on a shared centralized platform, documenting field updates, flagged concerns, corrective actions, and financial records. This layered documentation framework has been put in place to ensure both real-time oversight and retrospective review, strengthening transparency and methodological integrity throughout the survey.

**10. Review meetings:**

State teams: Weekly virtual review meetings are conducted between central program coordinators and each state’s field team to discuss progress, address deviations, and clarify protocols. Any red flags or protocol violations are discussed directly with the FDCs involved, and appropriate explanations are sought. These meetings allow state teams to raise queries or request support. Any operational disagreement or QC concern is addressed through the tiered mechanism. Issues are first discussed between the Field Data Collection team and the central State/UT Coordinators or Program Coordinators, as necessary. If unresolved, they are escalated to the State Principal Investigators and, if needed, jointly reviewed by the State PIs and the Central PI for resolution.

Central team: The central team, including Principal Investigators, Program Coordinators, IT specialists, statisticians, the QC team, and administrative staff, holds weekly meetings. During these meetings, each state’s progress is reviewed in detail. Data anomalies, protocol deviations, and operational challenges are discussed. Corrective or preventive measures are identified and finalized in consultation with state teams. Depending on the nature and frequency of deviations, these actions include retraining sessions on specific modules, interviewing techniques, or field protocols. Disciplinary actions, if required, are also recommended for persistent intentional deviations or quality issues being observed despite retraining.

All-center meeting: A monthly virtual all-center meeting is held with PI/Co-PIs from all states/UTs to discuss common ongoing aspects of the survey. This includes the progress of the survey, the challenges, training programs, and administrative aspects that may require discussion and addressing.

### Ethical considerations

The National Mental Health Survey–2 (NMHS-2) received ethical approval from the Institutional Ethics Committee of the National Institute of Mental Health and Neurosciences (NIMHANS), Bengaluru, India. All participants were provided with a clear explanation of the study purpose, procedures, and rights; written informed consent was obtained from adults. For adolescents, written parental consent and adolescent assent were obtained. This manuscript specifically describes the Quality Control (QC) protocol of NMHS-2 and does not report survey methodology or survey outcomes. Approval Reference Number: NIMHANS/ 42^nd^ EC (BEH.SC.DIV.)/2023, dated 8.12.2023.

### Project timeline

Participant recruitment was started in October 2024 and is slated to be completed by July 2026, with the publication of results anticipated by October 2026

## Discussion

High-quality epidemiological data are essential for designing effective public health interventions and informing policy. In large-scale surveys, especially those spanning diverse geographic, linguistic, and cultural contexts, quality control (QC) plays a critical role in ensuring the validity, reliability, and consistency of the data collected [18].

The National Mental Health Survey of India – 2 (NMHS-2) is a landmark effort. It is among the largest epidemiological surveys of mental disorders globally, with a sample of approximately >250,000 individuals, including adults, adolescents, and children. More than 300 trained FDCs have been estimated to be undertaking this nationwide endeavor, especially when the survey is to be administered in 20 different languages across states and union territories. The main challenge for the QC protocol is to ensure a framework for NMHS 2 to be administered uniformly across the country despite significant variations in language, culture, and social norms, along with the disparities in trained personnel, mental health infrastructure, digital access, and logistics. To address this, the primary strategy has been to ensure a multi-layered, adaptive quality control protocol to oversee all aspects of the survey, from training and administration to field data monitoring. The strategy of having layered operational structures is not new and has been deemed essential for coordination, accountability, and maintaining consistency across different aspects, particularly in complex national studies [6].

High-quality training is foundational to ensuring data accuracy and overall reliability in large-scale epidemiological surveys [1]. This has been a critical priority for the NMHS-2, which has employed a structured, multi-tiered training program for the lay interviewers, uniformly implemented to ensure standardized skills and survey readiness across all states and union territories. The cascading model adopted, employed in the World Mental Health (WMH) Surveys, also facilitates structured knowledge transfer and has been shown to promote standardization and fidelity in the implementation of large, multisite epidemiological studies [3].

Another aspect is the effective field-level monitoring, which has been ensured in the NMHS-2, which is a cornerstone of quality assurance in large-scale epidemiological surveys [13]. Continuous monitoring of interviewer performance is known to reduce measurement error and interviewer-related variability [15,16]. The multi-layered safeguards are specifically aligned with international recommendations to detect and prevent data falsification in field surveys, such as those outlined by the AAPOR and ASA Task Force Report, which emphasize supervision, verification, and the integration of paradata as critical quality control elements [19]. The robust multi-tiered administrative framework of the NMHS-2, with its numerous checks and balances, is also expected to effectively coordinate, ensure accountability, and maintain consistency across various survey sites of the NMHS-2.

This paper addresses a notable gap in published, context-specific guidance on structured and replicable QC strategies in large-scale mental health surveys— particularly within resource-constrained settings [1,14,20].

The QC framework defined for the NMHS-2 has conceptual foundations in the general frameworks such as the WHO STEPS Surveillance Manual, Quality Control and Good Epidemiological Practice, and the Good Epidemiological Practice (GEP) guidelines developed by the German Society for Epidemiology. It has also incorporated best practices of other national surveys, such as the U.S. National Comorbidity Survey Replication (NCS-R), the European Health Interview Survey (EUROHIS), the Canadian Community Health Survey (CCHS), the Saudi National Mental Health Survey, and the Qatar National Mental Health Survey to ensure data reliability and consistency [4,5,21–23].

The QC protocol operates within several practical constraints. Differences in state-level administrative capacity, delays in escalating QC deviations, and device or software issues may affect consistency in implementation. Intensive QC procedures may also increase workload for FDCs. These challenges are addressed through regular support from the central team, adequate leave provisions for FDCs, the availability of backup devices, real-time 24x7 IT assistance, triangulation of information from multiple monitoring sources, and weekly review meetings. All QC deviations and corrective actions will be documented and reviewed post-survey to inform improvements for future national surveys.

The NMHS-2 has established robust quality control protocols to enhance data quality and minimize methodological flaws and data inaccuracies. The a priori documentation of these processes ensures transparency and strengthens the credibility of the survey’s findings. The findings of the survey will be crucial for developing effective public mental health policies and interventions both at the national and state levels in the coming decade.
